# A Smart Textile-Based Tactile Sensing System for Multi-Channel Sign Language Recognition

**DOI:** 10.3390/s25154602

**Published:** 2025-07-25

**Authors:** Keran Chen, Longnan Li, Qinyao Peng, Mengyuan He, Liyun Ma, Xinxin Li, Zhenyu Lu

**Affiliations:** 1 School of Electrical Engineering, Xi’an Jiaotong University, Xi’an 710049, China; cranechen@stu.xjtu.edu.cn; 2 College of Intelligent Systems Science and Engineering, Harbin Engineering University, Harbin 150001, China; longnan_li@hrbeu.edu.cn; 3 Bristol Robotics Laboratory, University of the West of England, Bristol BS16 1QY, UK; hy22029@alumni.bristol.ac.uk (Q.P.); mengyuan.he@liverpool.ac.uk (M.H.); 4 Hamlyn Centre for Robotic Surgery, Department of Mechanical Engineering, Imperial College London, London SW7 2AZ, UK; liyun.ma@imperial.ac.uk; 5 College of Textiles, Donghua University, Shanghai 200051, China; lixinxin@dhu.edu.cn; 6 School of Automation, South China University of Technology, Guangzhou 510641, China

**Keywords:** smart textiles, tactile sensing, wearable sensors system, sign language recognition, human–computer interaction

## Abstract

Sign language recognition plays a crucial role in enabling communication for deaf individuals, yet current methods face limitations such as sensitivity to lighting conditions, occlusions, and lack of adaptability in diverse environments. This study presents a wearable multi-channel tactile sensing system based on smart textiles, designed to capture subtle wrist and finger motions for static sign language recognition. The system leverages triboelectric yarns sewn into gloves and sleeves to construct a skin-conformal tactile sensor array, capable of detecting biomechanical interactions through contact and deformation. Unlike vision-based approaches, the proposed sensor platform operates independently of environmental lighting or occlusions, offering reliable performance in diverse conditions. Experimental validation on American Sign Language letter gestures demonstrates that the proposed system achieves high signal clarity after customized filtering, leading to a classification accuracy of 94.66%. Experimental results show effective recognition of complex gestures, highlighting the system’s potential for broader applications in human-computer interaction.

## 1. Introduction

Deaf individuals face significant communication challenges in daily life, often leading to feelings of loneliness, isolation, and frustration, especially among older adults with hearing loss. One major challenge for this community is achieving proper integration into mainstream society [1]. The hands are one of the most important parts of the human body, and are responsible for manipulating objects, interacting with them, and transmitting information in daily life. One of the most powerful forms of human expression and communication is sign language, a visual and gestural language used by deaf and hard-of-hearing individuals around the globe [2]. As the main way for deaf people to communicate with the outside world, sign language is indispensable in acquiring knowledge, communicating with hearing people, and improving quality of life. Therefore, sign language plays a crucial role in helping deaf people integrate into various aspects of society. As people gain a better understanding of individuals with additional needs, the focus on sign language has become more widespread.

Sign language recognition refers to the use of algorithms and techniques to recognize the resulting sequence of gestures and to elaborate their meaning in the form of text or speech, and its related recognition techniques can also be applied to other fields, such as smart home interactions, traffic police command recognition [3], human–machine interactions [4,5,6,7], and intelligent driving [8]. Gesture recognition has long been a focus in human–computer interaction research, mainly through pattern recognition algorithms [9] to analyze the meanings expressed by gestures, so as to achieve smooth and accurate interaction between humans and machines.

Vision-based gesture recognition and wearable sensor-based gesture recognition are currently two mainstream approaches in gesture recognition technology. The primary distinction between them lies in the methods and formats of gesture data acquisition. However, both approaches adopt deep learning models for data processing. This method allows computational models with multiple processing layers to learn and represent data with multiple levels of abstraction to mimic human brain mechanisms and capture the complex features of large-scale data. Machine learning models, including support vector machines, random forests, K-nearest neighbors, and logistic regression, have been employed in hand gesture recognition [10]. Deep learning models, particularly convolutional neural networks (CNNs), have also been developed and optimized over time, emerging as the most widely used techniques for classification [10,11,12]. Sakshi sharma [13] developed an improved convolutional neural network model, named G-CNN, which features a compact architecture that delivers high classification accuracy with fewer parameters while demonstrating robustness to transformations such as rotation and scaling. The G-CNN model outperforms traditional architectures, such as VGG-16, in both recognition accuracy and computational efficiency, achieving impressive accuracies of 99.96% and 100% on the ISL and ASL datasets, respectively.

Vision-based methods use at least one camera to collect gesture data. Specialized cameras and depth sensors can provide additional information relating to where the hands are with respect to the camera. Many studies have used the Microsoft Kinect gaming camera (Microsoft Corporation, Redmond, WA, USA), which has a built-in depth sensor with an infrared projector that triangulates the distance between a signer’s hand and the camera [14,15]. Z. Parcheta used the Leap motion sensor, which is an optical two-camera system with three infrared light-emitting diodes for hand tracking [16]. The Leap motion sensor and Kinect camera have also been used simultaneously to capture the motion of the hands from two viewpoints, leading to the successful recognition of sign language gestures [17,18]. The development of vision-based recognition technology is relatively mature, but it is subject to the constraints of equipment and environmental conditions, such as the position angle of the camera, the camera being blocked, and the light being too bright or too dark, which will affect the quality of the pictures taken by the camera, thus affecting the recognition accuracy. During the acquisition of hand keypoints, monocular cameras may fail to capture certain spatial information due to inter-finger occlusions. Such occlusions often act as impediments, constraining the potential for enhancement in recognition accuracy. In gesture recognition, fingers can easily block each other, objects can block hands, or parts can become nearly unrecognizable due to being overexposed or too dark [19].

To address these issues, wearable sensor-based technologies offer a stable and adaptable alternative that performs reliably across diverse environments. Gesture changes result from muscle contraction [20], tendon movement, vascular deformation [21], and bone displacement, all of which can be detected [22] through various sensing methods, including electronic, mechanical, acoustic, and optical. Inertial sensors and myoelectric signal-based recognition systems are particularly advantageous, as they are unaffected by ambient light and background variations, allowing for consistent and straightforward signal processing. Wei Wang [23] developed a smart wearable system comprising five arch-structured self-powered triboelectric sensors, a microcontroller with an embedded analog-to-digital converter (ADC), and an AI block to rapidly recognize the signal patterns of sign language of 26 letters. Such systems offer miniaturization, low power consumption, and high integration, making them cost-effective and ideal for applications like medical rehabilitation robotics, sign language classification, and human activity recognition in complex environments.

Smart textiles, unlike traditional rigid materials, conform closely to the skin and integrate conductive fibers that transmit electrical signals to flexible components like sensors and chips [24]. Their flexibility and skin-friendly nature make them ideal for continuous wear, particularly in sign language recognition, where comfort is crucial. Smart textiles provide a non-intrusive way to monitor gestures while seamlessly integrating with other electronic components, ensuring effective gesture recognition without compromising user comfort. Myungsun Park [25] developed a glove with a soft, stretchable sensing layer composed of liquid metal sensors, which was used to collect a large dataset of natural hand motions for accurately quantifying bone lengths and joint angles. After gathering the hand motion data, the system employed a forward kinematics model to reconstruct hand poses. Luo et al. [26] integrated haptic sensors and vibrotactile actuators using digital embroidery for the capture, transmission, and sharing of haptic interactions. These gloves were used to alleviate tactile occlusion, guide users in physical skill training, and provide feedback in remote robot manipulation. The current limitation of these studies is that they only focus on tactile interactions with the hand or some fingers, as humans are most sensitive to tactile feedback from the hand, which limits the application of the system to other body parts.

Inspired by the above research, this research primarily designed and implemented a multi-channel sensing system using smart textile materials for sign language recognition. Key innovations of the work include the following:

- System Development: We developed a wearable multi-channel sensing system using smart textile materials, featuring conductive yarns that effectively capture subtle electrical signals. The innovative design ensures stable signal acquisition despite challenging conditions, while the flexibility and skin-friendly properties of the materials enhance adaptability and user comfort.
- Verification Approach: We processed and analyzed wrist and finger data separately using denoising techniques and dynamic threshold extraction. Experimental results demonstrated that our methods significantly improved signal recognition, achieving a classification accuracy of over 90%, thereby verifying the system’s effectiveness in recognizing complex gestures.

This paper is structured as follows: Section 2 introduces the system design, including the design and fabrication of smart textiles and experimental equipment; Section 3 introduces the data acquisition and processing methods; Section 4 presents the results of the data processing and classification experiments; and Section 5 summarizes the research results and proposes future directions for improvement.

## 2. System Design

**Figure 1:**
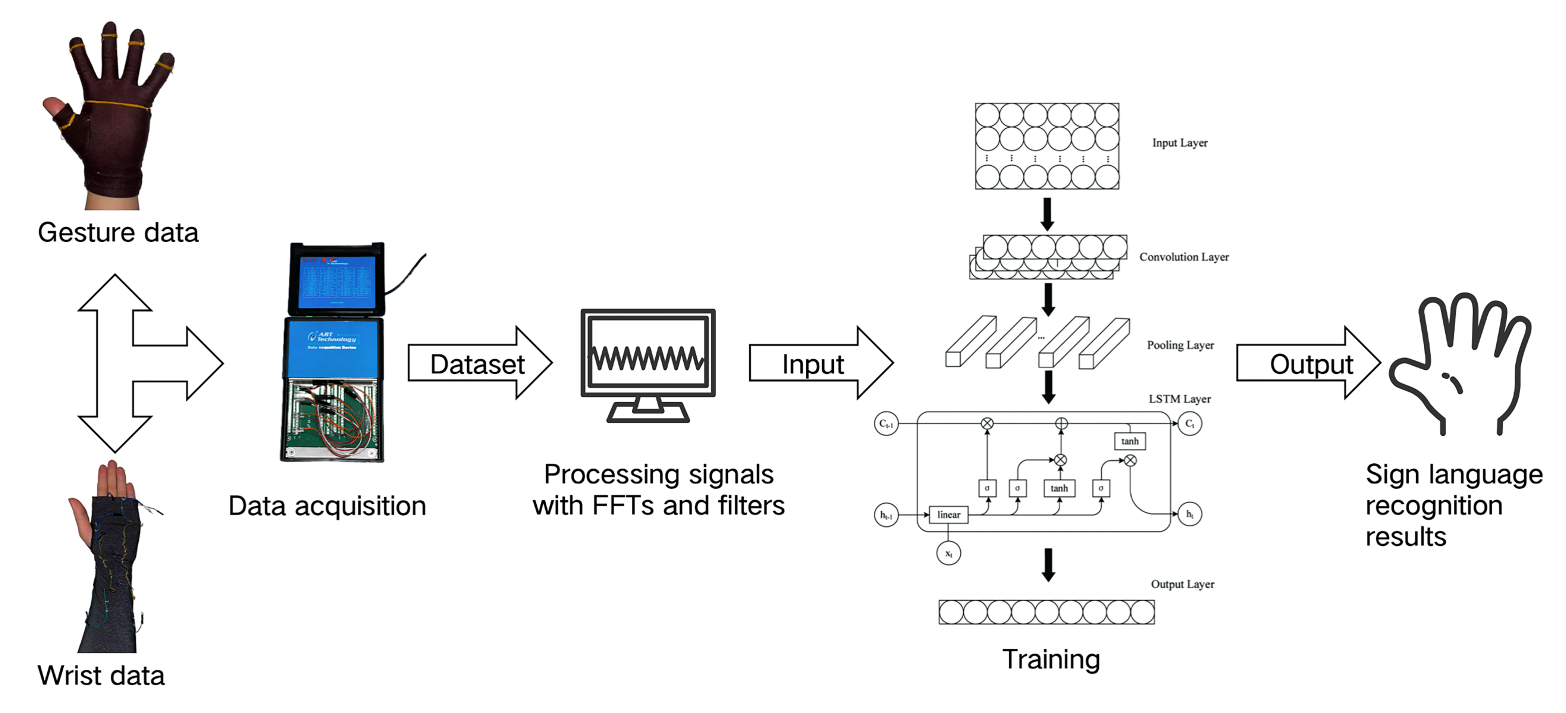
The structure of the system.

As shown in Figure 1, the entire data acquisition system consists of the following hardware components: a cotton glove and ice sleeve (both made of cotton and purchased via Amazon); a flame-retardant triboelectric yarn (FRTY) fabricated using a self-designed yarn wrapping setup; DuPont connecting cables; a USB-5633 data acquisition card; a Leap Motion; and a computer. The software portion of the system used the companion data acquisition application for the USB5633. The yarn was sewn at 90° intervals around the wrist area of the sleeve; on both the tip and dorsal sides of each glove finger; and at the center of the palm. The ends of each yarn were connected to the data acquisition card through DuPont cables, which were connected to the computer through a network cable. After the parameters are set in the signal acquisition software accompanying the USB5633, the system can start the data acquisition process. The acquired data is divided into wrist data and hand gesture data, and the former and the latter are processed by a VME or Butterworth filter, respectively, and the processed data is trained by machine learning.

### 2.1. Smart Textile Materials

A triboelectric nanogenerator (TENG) converts mechanical energy into electrical energy through contact electrification and electrostatic induction. When two materials come into repeated contact and separation due to external mechanical stimuli, electrons are transferred between their surfaces according to their triboelectric polarity, resulting in the accumulation of opposite charges. The subsequent separation of these materials creates a potential difference, which drives transient displacement currents through an external circuit. If the materials are separated by additional mechanical energy, the charge creates a potential difference between the conductive materials, causing the charge to move from one side to the other. Wearable TENGs are becoming more attractive every day, especially in the areas of human motion tracking, identification, health monitoring, and human–machine interfaces. For TENGs, all the selected materials need to possess the essential properties of flexibility, durability, and ductility, as the goal of any TENG is to be successfully incorporated into a garment.

Recent research [27] has also demonstrated that triboelectric yarns can maintain stable output characteristics even under thermally demanding or mechanically dynamic conditions, highlighting their robustness as a platform for wearable sensing. In particular, the use of flame-retardant textile substrates has drawn growing attention in triboelectric nanogenerator (TENG) design due to their inherent thermal stability, structural integrity, and safety advantages. By combining polymeric insulators with thermally inert textile scaffolds, it is possible to construct triboelectric systems that preserve surface charge interactions under varying mechanical strain or environmental stress. These material-level considerations are especially critical for applications in wearable electronics, where fabrics are exposed to continuous deformation, heat from the human body, or even high-temperature operating environments. The integration of such triboelectric yarns into garment systems not only enhances sensing stability but also aligns well with the demands of durability and user comfort in long-term deployment.

**Figure 2:**
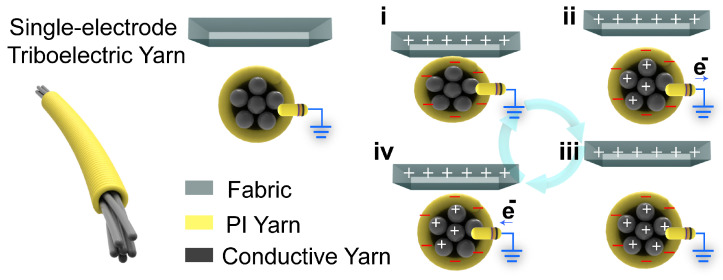
Yarn structure and working theory (i–iv).

The flame-retardant triboelectric yarn (FRTY) used in this study is a typical TENG. The yarn is a mechanically manufactured, three-dimensional honeycomb-structured, flame-retardant friction-generating fabric. The composite yarn is produced using continuous hollow spindle fancy twister technology, where each single yarn is a single-electrode triboelectric yarn (SETY), enabling it to perform sensing functions. The working mechanism of the SETY follows the single-electrode mode, whose working principle is schematically shown in Figure 2. The materials selected for the charge-generating layer of any TENG are required to either hold a tribo-positive or tribo-negative charge, the significance of which directly effects the overall output of the device [28]. In this study, the FRTY utilizes polyimide (PI) yarn as the triboelectric material with a negative charge tendency, fabric as the triboelectric material with a positive charge tendency, and metal-infused conductive yarn as the electrode. This yarn material, capable of transmitting electrical signals, can be seamlessly integrated into clothing and wearable devices to capture motion data without interfering with natural movement.

When skin comes into contact with the FRTY, the FRTY acquires a negative charge due to the greater ability of the PI yarn to acquire a negative charge. Once the relative separation occurs, the external negative charge induces a positive charge to flow from the poles to the earth. When the skin is completely separated from the material, the electrons in the FRTY are balanced and electron flow no longer occurs. Thereafter, when the skin approaches the FRTY again, the opposite electron flow occurs from the ground to the electrodes until the skin is once again in close contact with the FRTY. By repeating the process of contact and separation, the FRTY will generate an alternating output current.

### 2.2. Design and Fabrication of Experimental Equipment

In order to measure as many hand gestures as possible, the location of the sensor needs to be precisely designed. FRTY material is an ideal sensor material because of its softness and good electrical conductivity, which outputs an alternating current (AC) electrical signal during the repeated contact and separation of its different layers. In this study, FRTY material is used as a sensor to collect data from various hand movements. Therefore, our main task is to ensure that the material is in close contact with the human body while allowing a sufficient range of motion. The hand movements involved in everyday gesture expressions are analyzed by referring to the alphabet in the American Sign Language manual. The sign language movements are classified into four categories: finger bending, wrist movement, fingertip touching, and palm interaction. The related movements require sensors (FRTY) at different locations for recognition.

**Figure 3:**
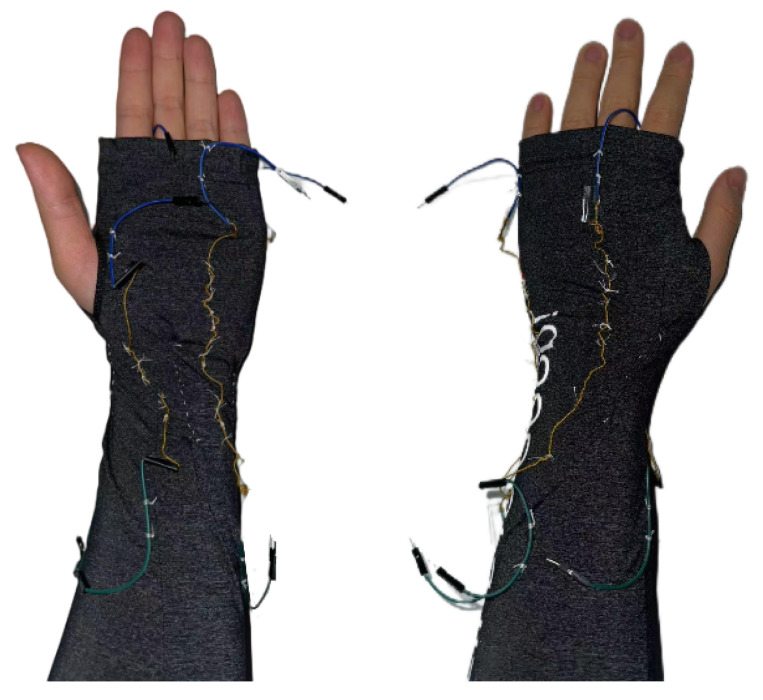
Sewing the yarn onto the sleeve.

We chose the ice sleeve as the platform for a device that collects wrist movements because of its ability to completely cover the human limb from elbow to hand and its superior yarn-holding ability, providing stable support and minimal friction over a wide range of movements. In addition, since the conductive material inside the yarn is easily separated from the insulating yarn wrapped around it and the material tends to break after repeated movements, we used ordinary non-conductive cotton thread instead of yarn to complete the fixation in subsequent experiments. This greatly reduced the risk of circuit interruption due to material breakage. After comparing the results of different forms of testing, the final choice for this experiment was to fix the yarn to the sleeve in a relatively loose manner at even intervals, as shown in Figure 3. At this point, the material can better fit the wrist and collect a stable electrical signal with the movement of the wrist.

**Figure 4:**
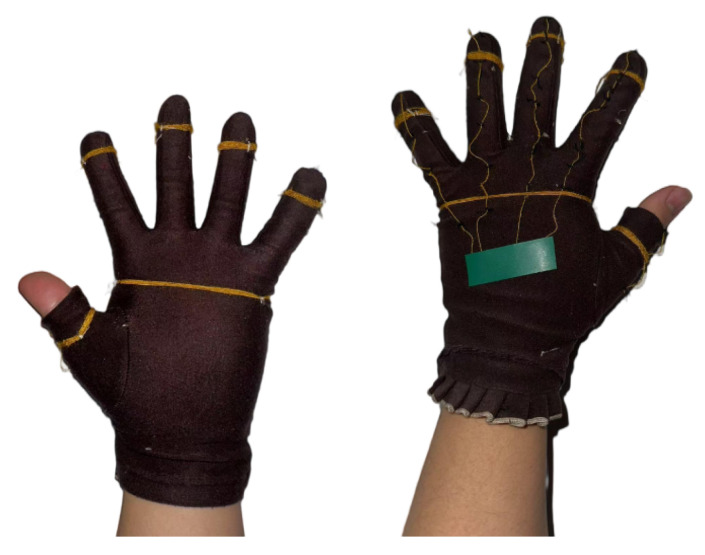
Sewing the yarn onto the glove.

To collect data on finger bending, fingertip touch, and palm interaction, it is necessary to use wearable gloves integrated with sensors. It has been observed that FRTY yarn generates a significant voltage signal when bent or in contact with the skin. Therefore, the design involves wrapping the yarn around the most frequently contacted areas of the fingers and palm, specifically the first knuckle of each finger and the center of the palm, to detect fingertip touch and palm interaction. Additionally, the yarn is sewn onto the glove along the dorsal side of each fingers from the fingertips to the metacarpals to capture finger bending movements. As shown in the Figure 4, the gloves are made of cotton fabric, with a portion of the fabric at the first knuckle of the thumb removed to ensure exposure of the thumb’s first knuckle when worn, while the rest of the glove remains intact. The yarn is evenly wound around the index finger, middle finger, ring finger, little finger, the base of the thumb, and the central area of the palm, with six to seven turns to ensure sufficient contact area with the skin. The ends of each group of yarns are twisted together with DuPont wires and secured with electrical tape to ensure a robust connection.

### 2.3. Hardware and Software Design

The system is designed to ensure that the first four sets of electrical signals (wrist signals) collected via USB5633 are accurately compared with the gestures and motion amplitudes collected by Leap Motion, while avoiding interference between the six sets of hand signals to ensure data accuracy. Therefore, in the envisaged scenario, time differences or delays between the two data collection processes should be avoided as much as possible. According to preliminary experimental tests on the two sets of collected data, the current time difference is within an acceptable range. After the signal is collected, the data will be processed and used for training appropriately.

This subsection will detail the hardware selection and software configuration used in the experiment to meet the needs of the experiment.

#### 2.3.1. Hardware

- USB5633: The work employs the USB5633 data acquisition device, developed by Beijing Altai Technology Development Co., Ltd., China, for voltage collection. During the data acquisition and system application process, it is necessary to collect data from 15 yarns located at various parts of the palm and wrist. To avoid signal interference from different channels, DuPont wires were used to establish connections between the signal acquisition card and the yarns. These connection structures underwent extensive testing to ensure optimal signal integrity.
- Leap Motion: Leap Motion is an advanced device designed for the precise capture and tracking of hand movements, supported by proprietary software. Manufactured by Leap Motion Inc. (San Francisco, CA, USA), it is employed in this experiment to collect wrist motion amplitudes and angles at a sampling rate of 10 Hz with minimal latency, aligning these measurements with the voltage signals generated by the triboelectric yarn. In this study, the device is used exclusively to output wrist angle information for constructing the wrist motion dataset; it is not involved in the subsequent training process or in the practical deployment of the recognition system.

#### 2.3.2. Software

The software design for this study integrates various signal processing and classification methods to enhance data acquisition and analysis. Using the USB5633 software (V6.00.00) application, configured for continuous Analog Input (AI) and finite-point sampling, the system precisely collects, controls, and organizes gesture data. The finite-point sampling function enables consistent sampling across known time windows, while object-oriented programming facilitates device control and data management through a structured interface.

To process these signals, a Fourier transform decomposes complex signals into sine wave components for effective frequency domain analysis. For noise reduction, a Butterworth filter was employed due to its flat response in the passband, minimizing signal distortion. Additionally, VME was used to separate and extract specific signal modes, ensuring a high signal-to-noise ratio.

For classification, SVM was applied to the wrist data to leverage its robust boundary maximization, enhancing classification accuracy. The CNN-LSTM model was used to classify multi-channel time-series signals from hand gestures, benefiting from neural networks’ ability to learn complex spatial patterns and avoid reliance on hand-crafted features. The combined use of these advanced signal processing and machine learning techniques allows for precise and reliable gesture recognition.

## 3. Experimental Approach

The next sections describe the experimental scenarios and the experimental process in detail, explaining how to configure these hardware devices and their structure, software parameters, and algorithms, and justify the choice of these methods.

### 3.1. Data Acquisition

For wrist data, the experimental setup involves limited-point sampling, utilizing four sampling channels, selecting pins compatible with the acquisition card, with a total runtime of 5 s. Each experiment begins with the arm held horizontally still until Leap Motion data stabilizes. Gestures are measured simultaneously using USB5633 and Leap Motion, outputting electrical signals and wrist angle information.

**Figure 5:**
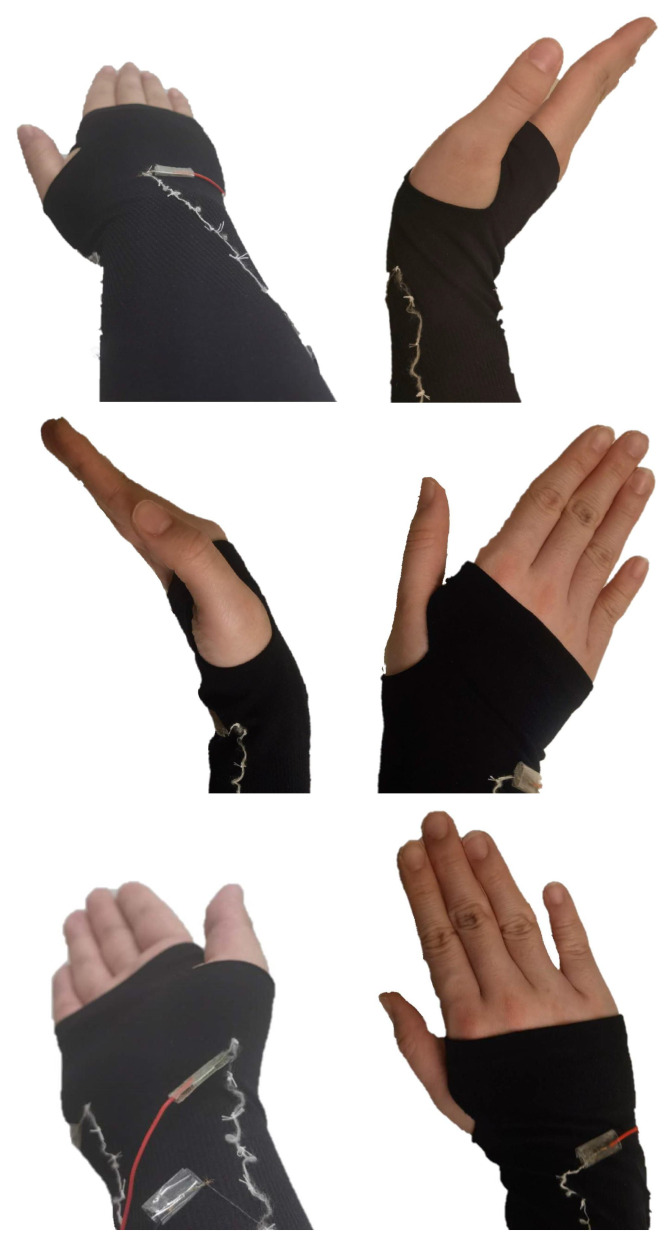
Wrist gestures collected by wearing data collection sleeve.

A total of six different wrist gestures were captured in the experiment, as shown in Figure 5. Leap Motion outputs two data columns, with the initial angle as the reference point, the first column representing the angle of the wrist swinging back and forth, and the second column representing the angle of swinging left and right. At the same time, the USB5633 software measures the electrical signals from wrist motion and uses a python script to match the voltage value on the yarn at the current moment with the wrist angle measured by the Leap Motion every 0.1 s and outputs it in binary form, ultimately forming a wrist motion dataset.

To collect gesture data, the sampling rate was set to 3000 samples per second, with a total of 15,000 sampling points, a signal gain of 8 times, a wiring mode of “end-to-ground,” and a signal range from −5 V to 5 V. After placing the gloves on the hands, they were adjusted to a comfortable position. Then, the DuPont wires were connected at the ends of the yarns to the corresponding channels of the data acquisition card. Sequentially, each group of yarns was touched with the skin and the fingers were bent to confirm the proper functioning of the sensors.

**Figure 6:**
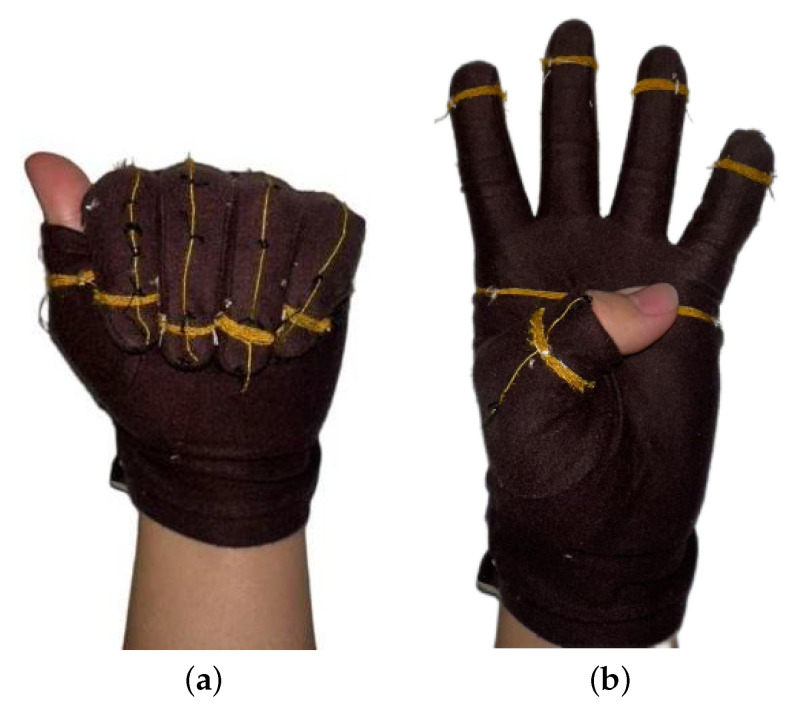

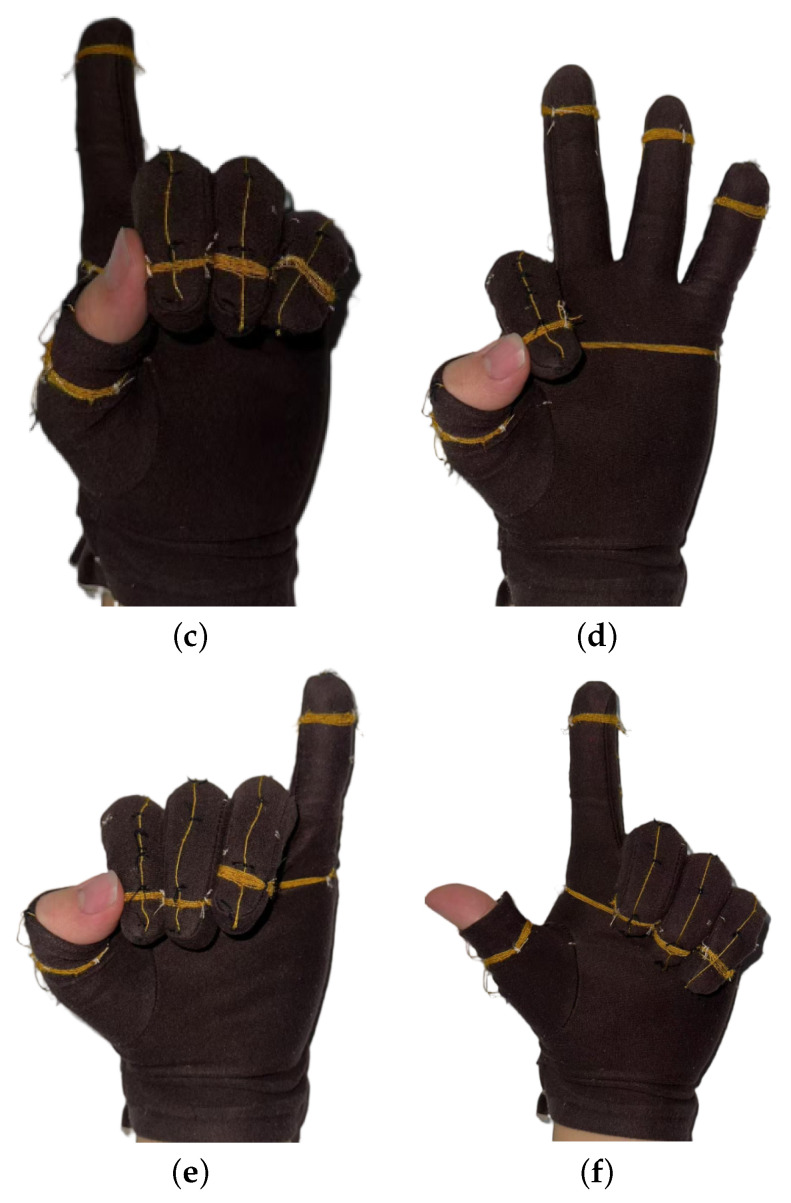
Selected letter gestures collected while wearing data collection gloves: (**a**) A in American Standard Sign Language (ASL), (**b**) B in ASL, (**c**) D in ASL, (**d**) F in ASL, (**e**) I in ASL, (**f**) L in ASL.

The reference gestures were based on the 24 letters from the American Sign Language manual, excluding “j” and “z,” as these involve dynamic sign recognition, which the current system is unable to distinguish from other letter gestures. In this process, “Start Single Limited Point Acquisition” is clicked to complete a set of hand movements within five seconds. Taking the letter “a” as an example, the forearm is raised with the palm facing forward, the thumb is extended, and the remaining four fingers are curled against the palm to complete the gesture. The gestures for other letters follow the same principle. To achieve optimal classification performance and eliminate random errors, sufficient data is required to train the model. Therefore, each letter gesture was repeated thirty times. After sampling, the binary data files were named according to the action number and repetition count. The above steps were repeated until all data measurements were completed. Some of the letter gestures included in the dataset are shown in Figure 6. Both the wrist dataset and the gesture dataset were divided into training and testing sets in a 4:1 ratio, with 80% of the data used for training and 20% for validation.

**Figure 7:**
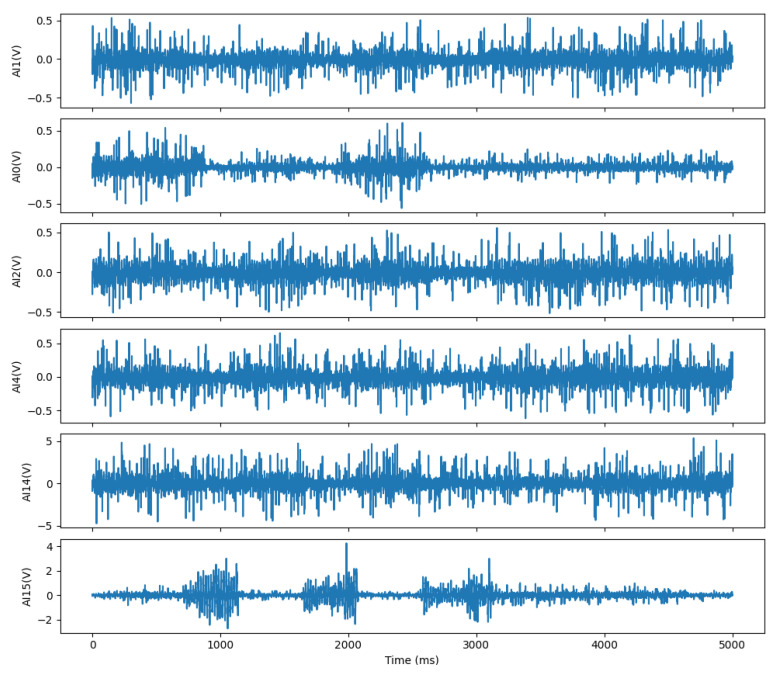
Hand gesture signal waveform.

As shown in Figure 7, after the binary bin file collected by the system software is converted into a waveform diagram by the plotting program, it can be clearly seen that the gesture signal is mixed with a lot of noise interference. If this noise is not processed, it may significantly affect the training effect of the subsequent neural network model, resulting in a decrease in the classification accuracy. The next subsection will describe in detail the processing method of the dataset and how to input the processed data into the neural network model for training.

### 3.2. Data Processing

The wrist data undergoes denoising processing via VME to improve the signal-to-noise ratio. Dynamic thresholds are obtained through Hilbert transform processing of electrical signal data to better adapt to signal changes. Then, feature extraction is performed on the processed data to retain signal patterns and differentiate between different gestures. The experiment utilizes Mean Absolute Value, Root Mean Square, and Autoregressive model coefficients to model different surface sEMG signals, which are then inputted into the network for classification.

FFT is applied to *N* sampling points, where *N* is typically set to a power of 2 to optimize computational efficiency. The pre-conditions of the FFT algorithm include the sampling frequency Fs, the signal frequency *F*, and the number of sampling points *N*. After the FFT transform, the result is a vector set containing *N* complex numbers, each of which corresponds to the amplitude and phase characteristics of a frequency point.

Specifically, the modulus value of each frequency point returned by the FFT represents the amplitude of the signal at that frequency, while the phase value reflects the phase of the signal at that frequency. If the peak value of the original signal is denoted by *A*, then the amplitude at each frequency point (except the first point representing the DC component) is approximately 2×A/N. The first point corresponds to the DC component (i.e., the component with zero frequency), while the (N+1)th point corresponds to the sampling frequency $F_{s}$. The N−1 points between them are equally spaced across the frequency range, with a step size of $F_{s}/N$. Therefore, the frequency of the *n*th point is:

(1) $$F_{n}=\left(n-1\right)\times \frac{F_{s}}{N},$$

where $F_{n}$ denotes the frequency represented by the nth frequency point, and the resolution is $F_{s}/N$. The frequency resolution is inversely related to the sampling time, and an increase in the number of sampling points will directly improve the frequency resolution.

**Figure 8:**
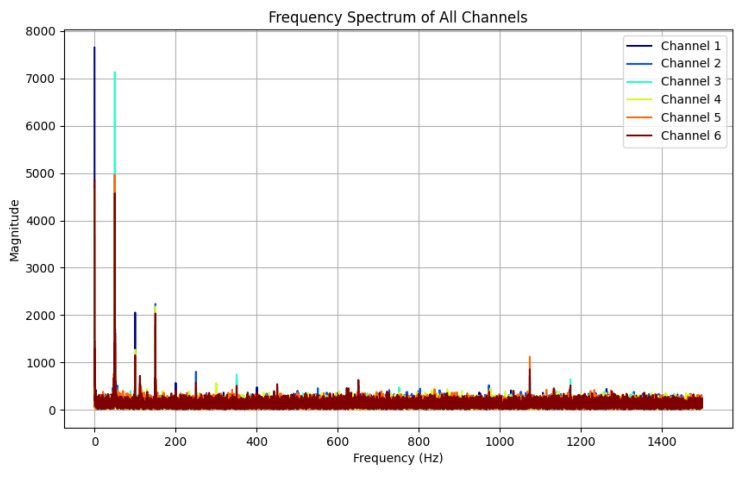
Spectrogram of the timing signal produced by S1–S6 yarns.

By applying Fast Fourier Transform (FFT) to one randomly selected piece of data from each category in the hand gesture dataset, the corresponding spectral analysis results can be obtained, and the spectra of all the signals can be displayed in the same graph. A gesture can be selected in relation to the spectrum diagram, as shown in Figure 8.

The results show that in the low-frequency band from 0 Hz to 400 Hz, the signal channels with significant frequency peaks are consistent with their labels, i.e., the thumbs touched the yarn loops of the corresponding channels during the data acquisition cycle, generating electrical signals. In the high-frequency band, on the other hand, no significant frequency peaks or active channels were observed, which indicates that the gesture signals were mainly concentrated in the low-frequency region, whereas the noise appeared mainly in the mid- and high-frequency regions. This result provides a basis for the parameter selection of the Butterworth filter, especially the cut-off frequency setting of the low-pass filter.

The Butterworth filter is a type of signal processing filter [29] designed to make the frequency response as flat as possible in the passband, i.e., free of any fluctuations. It is also known as a maximum flat amplitude filter. A distinctive feature of this filter is that the amplitude–frequency characteristics are smoothest in its passband range, thus not introducing any ripples or artefacts. It was applied to all the data in the dataset and the performance of the Butterworth filter was measured using MSE [30] and SNR before and after filtering as metrics.

Based on the data resolved after spectral analysis of the signal, the cut-off frequency of the Butterworth filter is set to 400 Hz and the order is set to third order. The high-frequency noise signal is almost completely eliminated after filtering, while the hand gesture signal in the low-frequency range is well preserved. This shows that the Butterworth filter can effectively remove high-frequency noise while maintaining the key characteristics of the hand gesture signal.

**Figure 9:**
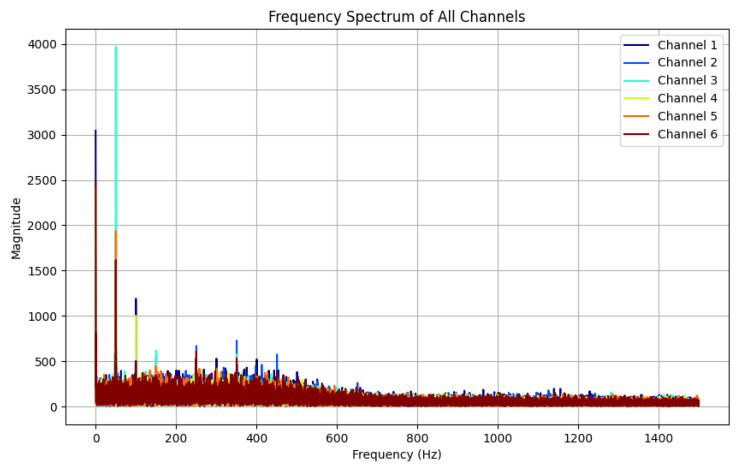
Spectrogram of the filtered timing signal generated by S1–S6 yarns.

The signal in Figure 8 was processed using a Butterworth filter, and the spectrum of the signal before and after filtering was analyzed using an FFT to verify the performance of the filter. Figure 8 shows the spectrum before filtering, and it can be seen that there is obvious noise and interference in the high-frequency range. In contrast, Figure 9 shows the result after processing by the Butterworth filter. The high-frequency noise signal is almost completely eliminated after filtering, while the hand gesture signal in the low-frequency range is well preserved.

SNR is used to evaluate the effectiveness of the filter in removing noise and retaining the useful signal. The higher the SNR, the greater the proportion of signal relative to the noise, and the more effectively the filter removes the noise while retaining the useful portion of the signal. The formula is as follows:

(2) $${\mathrm{SNR}}_{\mathrm{dB}}=20\log_{10}\left(\frac{V_{\mathrm{signal}}}{V_{\mathrm{noise}}}\right)$$

MSE is used to measure the error between the signal processed by the filter and the original signal. The smaller the MSE is, the closer the filtered signal is to the original signal, i.e., the filter removes the noise while retaining as much as possible the characteristics of the useful signal. The formula is as follows:

(3) $$\mathrm{MSE}=E\left[{\left(d\left(n\right)-y\left(n\right)\right)}^{2}\right]$$

### 3.3. CNN-LSTM Model Building

Sign language recognition, as a sequential task, heavily relies on the quality of neural network modeling, which is a critical factor determining the recognition performance. The core structure of a convolutional neural network (CNN) lies in its convolutional and pooling layers. By leveraging convolutional layers with varying numbers of channels and pooling layers, the network extracts as many features as possible, minimizing the need for manual feature engineering. Through supervised learning, the computational power is fully utilized to actively identify suitable feature representations. The convolutional layer, being the core of the CNN, employs convolutional kernels (filters) that slide over the input data, performing local operations to extract local features such as edges, textures, and trends. The pooling layer is used to downsample the feature maps output by the convolutional layer, reducing their dimensions, lowering computational complexity and the number of parameters, and preventing overfitting.

Long Short-Term Memory (LSTM) networks are designed to address the long-term dependency issues that traditional Recurrent Neural Networks (RNNs) struggle with. The inclusion of forget gates and modifications to the network structure effectively mitigate the problems of vanishing and exploding gradients that are common in many RNNs. LSTM utilizes memory cells, input gates, forget gates, and output gates to determine which information to retain and which to discard, capturing long-range dependencies in the data to generate more global feature representations.

However, both CNN and LSTM models have their limitations. The CNN-LSTM model adopted in this study integrates the feature extraction capabilities of CNN with the sequential modeling strengths of LSTM, enabling more efficient capture of both temporal and spatial features within the data. This integration significantly enhances the model’s performance in tasks such as sequential data processing, image processing, and video analysis.

**Figure 10:**
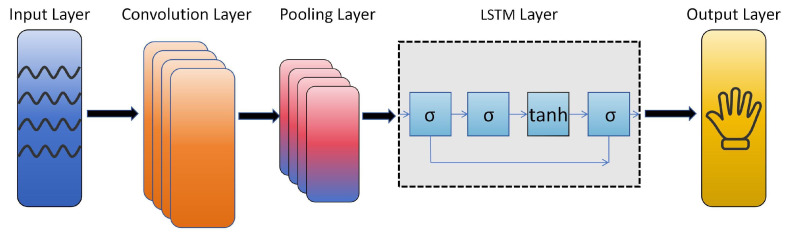
The structure of the CNN-LSTM model.

The architecture of the multi-feature sequence classification CNN-LSTM model is illustrated in Figure 10. It primarily consists of an input signal layer, CNN convolutional layers, pooling layers, LSTM layers, and a classification output layer. The process of the multi-input sequence classification task based on CNN-LSTM is as follows:

1. The input signals are normalized and fed into the CNN convolutional layers, where wide convolutional kernels adaptively extract features;
2. The extracted features undergo pooling operations in the max-pooling layers to reduce data dimensionality while retaining the primary feature information;
3. The dimensionality-reduced feature data is then fed into the LSTM layers as input features, enabling the neural network to automatically learn sequential characteristics;
4. The Adam algorithm is used to propagate the training errors backward, updating the model parameters layer by layer;
5. The Softmax activation function is applied to classify the signal features, completing the multi-feature input sequence classification task.

## 4. Experiments

### 4.1. Results of Data Processing

**Figure 11:**
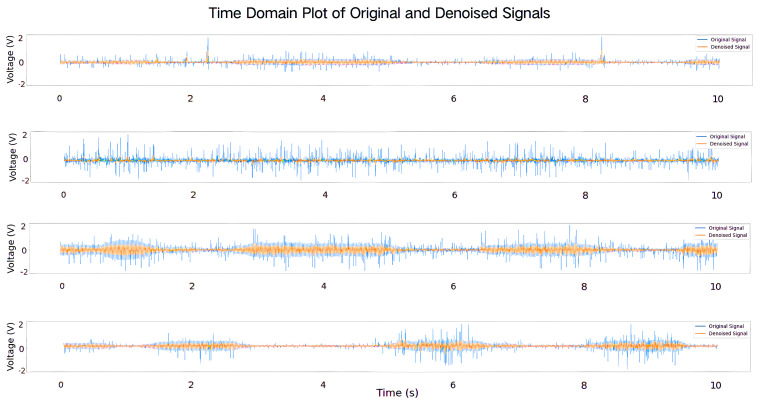
Before and after comparison of the wrist signal processed by VME.

As shown in Figure 11, the electrical signal curves after denoising processing by VME are depicted. The horizontal coordinate indicates the number of seconds, and the vertical coordinate indicates the voltage value. Taking the example of rapid back-and-forth wrist movements, the blue curves in the graph represent the initial signals, while the orange curves represent the data after denoising processing. The results indicate that VME effectively removes noise, aiding in a more accurate analysis and identification of the target signal.

The metrics before and after filtering for one of the selected data points are shown in Table 1.

From the above metrics, the following can stated:

- All channels have negative SNR before filtering, indicating that the noise is stronger than the signal before filtering and the signal is almost drowned by the noise. A negative SNR value means that the signal quality is very poor and noise dominates the signal. After Butterworth filtering, the SNR is significantly improved, indicating that the filter effectively preserves the signal and reduces the effect of noise.
- The mean square error (MSE) values of all channels are below 0.001, indicating that the error between the filtered signal and the original signal is very small. The lower the MSE, the closer the filtered signal is to the original signal, and the noise is effectively removed while the useful signal components are retained better without introducing significant distortion.

### 4.2. Results of Classification

Through analysis of the experimental results, it was found that the electrical signals generated by the yarn vary with different wrist positions, helping to distinguish between different gestures. The differences in electrical signals are related to the direction and magnitude of wrist movement. Among the four tested directions, the signals of the two swinging directions oscillate more significantly, with larger swinging amplitudes corresponding to faster wrist swinging rates. When swinging side to side, the signal fluctuations on the left and right sides of the wrist are more pronounced, while swinging forward and backward results in larger amplitude curves on the front and back sides. The experiment matches the classification results with the gesture data obtained from Leap Motion as visual data for the electrical signals. Finally, feature classification is performed, and the KNN algorithm is utilized to compare the average recognition rates of six different gestures under different K values. The results of the classification of the wrist data are shown in Table 2. When K = 31, the recognition rate is the highest, at 88.28%. The recognition rate achieved by the SVM algorithm is 5.58% higher than that obtained by the KNN algorithm, and it has a faster classification speed. The classification times during training, shown in Table 2, were 7.38 and 5.35 s; this only reflects the training phase and does not impact real-time usage. After training, the system can classify a single wrist movement within just a few milliseconds, making it well-suited for practical applications in sign language recognition.

**Figure 12:**
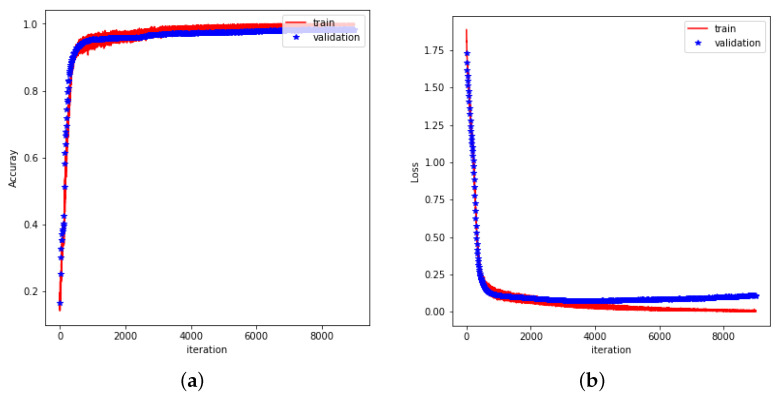
(**a**) Accuracy during training of CNN-LSTM. (**b**) Loss rate during training of CNN-LSTM.

The flexibility of neural network structures allows for the formulation of various tasks, whether they are classification problems, regression problems [31], or a combination of both [32]. CNN-LSTM was used to train and validate hand gesture data, with model performance evaluated by training accuracy, validation accuracy, training loss, and validation loss. As shown in Figure 12a, the training accuracy and validation accuracy of the model increase rapidly at the beginning of training, gradually converge, and converge to 1.0 (i.e., close to 100%) with the increase in epochs. This indicates that the model exhibits high classification accuracy on both training and validation data.

The accuracy of the model on the training and validation sets is almost identical, indicating that the model not only fits the training data well, but also maintains the same high accuracy on the validation data. The difference between the training and validation accuracies is extremely small, indicating that the model does not overfit on the training set and has good generalization ability. The performance of the loss function is shown in Figure 12b. The training loss and validation loss decrease rapidly at the beginning of the training period and level off at a later stage, eventually remaining at a low level. This further validates the effective learning of the model during the training process.

Within the first few epochs of training, the loss function decreases rapidly, showing that the model is able to effectively learn the features of the data and reduce the prediction error; as the training proceeds, both the training loss and the validation loss tend to stabilize and the difference between them is not significant, which indicates that the model maintains a stable learning effect during the training process. The validation loss did not appear to be significantly higher than the training loss, indicating that the model did not show significant performance degradation on the validation data and was able to handle unseen data better.

These results indicate that the trained CNN-LSTM model is able to successfully capture the key features in the gesture data and has good generalization ability for gesture recognition tasks. The accuracy of the CNN-LSTM model on the test set is 94.66%.

In order to better reflect the advantages of the CNN-LSTM model in the task-heavy classification of temporal signals, the LSTM model is selected as the baseline model for comparison. The same sign language dataset is input into the LSTM model and the training results obtained are as follows.

**Figure 13:**
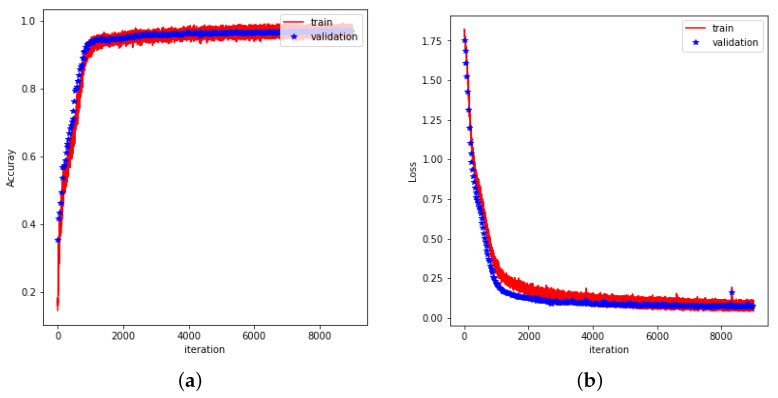
(**a**) Accuracy during training of LSTM. (**b**) Loss rate during training of LSTM.

Figure 13a shows that LSTM training and validation accuracy initially increase rapidly, and then stabilize close to 1.0. This indicates that the LSTM model is able to effectively capture patterns in time-series data and is successfully used for the gesture recognition task.

The accuracies of the training and validation sets almost overlap, indicating that the model has consistent performance on both training and validation data, which suggests that the LSTM model has no overfitting phenomenon and has strong generalization ability.

As shown in Figure 13b, the loss function decreases rapidly in the early stage of training, indicating that the LSTM model is able to learn the data features effectively in the early stage, and the prediction error is reduced substantially. With the increase in the depth of training, both the training loss and the validation loss tend to be stable, and the two curves are close to each other, indicating that the model maintains good stability in the learning process, and there is no overfitting phenomenon. The accuracy of the LSTM model on the test set is 89.87%.

As shown in Table 3, the CNN-LSTM model marginally outperforms the standalone LSTM model in gesture recognition tasks, particularly in scenarios requiring spatial feature extraction. The convolutional layers of CNN, as employed in this study, demonstrate superior capability in capturing localized spatial patterns, rendering them highly effective for processing multi-channel sensor data. The CNN-LSTM architecture supports a multi-branch CNN structure, where features from distinct modalities are independently extracted by convolutional operations before being fused through LSTM for temporal modeling. This structural advantage enables the hybrid model to achieve enhanced classification accuracy by efficiently leveraging both spatial correlations across sensor channels and temporal dependencies in sequential data.

Although the standalone LSTM model achieves a stable accuracy of 86.32% and its recurrent architecture effectively captures temporal dependencies, the gesture patterns investigated in this study exhibit limited demand for long-term temporal relationships while benefiting more dominantly from spatial feature representation. Furthermore, the dimensionality-reduced features generated by CNN layers serve as optimized inputs to the LSTM module, which reduces the temporal sequence length and hidden layer dimensionality compared to processing raw sensor data directly. Consequently, the CNN-LSTM model demonstrates superior computational efficiency and classification performance in this task, suggesting that architectures emphasizing spatial feature extraction are more suitable for multi-channel sensor-driven scenarios characterized by strong inter-channel spatial correlations.

## 5. Conclusions

This study presented a multi-channel sensing system based on smart textiles for hand gesture recognition, utilizing conductive yarns and data acquisition modules to detect subtle electrical signals from wrist and finger movements. The system demonstrated the benefits of smart textiles in providing a comfortable, wearable, and non-invasive solution that maintains stable performance even in challenging environments, such as those with uneven lighting or obstructions. Through the use of distinct processing techniques for wrist and finger data, the system attained a high level of classification accuracy. These results validate the system’s effectiveness for sign language recognition and suggest potential applications in broader human–computer interaction scenarios.

However, the system still exhibits several limitations. The current sign language recognition system suffers from significant latency, as gesture recognition is performed only after data acquisition is completed, which restricts its applicability in real-time scenarios. The reliability of TENG materials in high-frequency daily use has not yet been validated. When contaminated by liquids or experiencing internal core fractures, the recognition accuracy can be severely compromised. Moreover, since the material is sewn on the fabric, it is challenging to remove and replace. Additionally, the current dataset is limited to a subset of letters from American Sign Language, and the recognition capabilities for dynamic gestures, multi-letter words, and sentences remain to be developed.

Future work will address these limitations through several key improvements. Applying a waterproof and oil-repellent nano-coating, such as a fluorinated polymer or hydrophobic coating, to the surface of the TENG material ensures stability in humid or contaminated environments. Incorporating multi-sensor fusion [33], such as IMU sensors [34,35], will enable recognition of more complex gestures by integrating diverse data types. Expanding the dataset to include gesture combinations and overlapping actions will improve recognition capabilities for real-world scenarios [36]. Higher-resolution acquisition equipment can reduce signal noise, and deploying the model on local devices can optimize real-time recognition [37] and improve system responsiveness.

## Figures and Tables

**Table 1:** Improvements in SNR and MSE across some channels after filtering.

	SNR Before Filtering	SNR After Filtering	MSE
Channel 1	−5.00	30.00	0.001230
Channel 2	−7.00	28.50	0.001012
Channel 3	−6.50	29.00	0.001105
Channel 4	−4.75	31.00	0.000989
Channel 5	−6.25	29.50	0.001080
Channel 6	−5.50	30.50	0.000954

**Table 2:** The classification result of KNN and SVM.

Sort	Accuracy Rate	Training Time (s)
KNN	88.28%	7.38
SVM	93.86%	5.35

**Table 3:** The classification results of CNN-LSTM and LSTM.

Sort	Accuracy Rate
CNN-LSTM	94.66%
LSTM	89.87%

## Data Availability

The original contributions presented in this study are included in the article. Further inquiries can be directed to the corresponding author.

## Abbreviations

The following abbreviations are used in this manuscript:

AI	Artificial Intelligence
CNN	Convolutional Neural Network
ASL	American Sign Language
TENG	Triboelectric Nanogenerator
FRTY	Flame-Retardant Triboelectric Yarn
SETY	Single-Electrode Triboelectric Yarn
PI	Polyimide
FFT	Fast Fourier Transform
VME	Variational Mode Extraction
SNR	Signal-to-Noise Ratio
MSE	Mean Square Error
SVM	Support Vector Machine
LSTM	Long Short-Term Memory
RNN	Recurrent Neural Network
IMU	Inertial Measurement Unit
